# Transforming Growth Factor-β2 Downregulates Major Histocompatibility Complex (MHC) I and MHC II Surface Expression on Equine Bone Marrow-Derived Mesenchymal Stem Cells Without Altering Other Phenotypic Cell Surface Markers

**DOI:** 10.3389/fvets.2017.00084

**Published:** 2017-06-12

**Authors:** Alix K. Berglund, Matthew B. Fisher, Kristin A. Cameron, Emma J. Poole, Lauren V. Schnabel

**Affiliations:** ^1^Department of Clinical Sciences, College of Veterinary Medicine, North Carolina State University, Raleigh, NC, United States; ^2^Comparative Medicine Institute, North Carolina State University, Raleigh, NC, United States; ^3^Department of Biomedical Engineering, North Carolina State University, Raleigh, and University of North Carolina at Chapel Hill, Chapel Hill, NC, United States

**Keywords:** mesenchymal stem cell, equine, allogeneic, major histocompatibility complex, transforming growth factor-β2, IFN-γ

## Abstract

Allogeneic mesenchymal stem cells (MSCs) are a promising cell source for treating musculoskeletal injuries in horses. Effective and safe allogeneic therapy may be hindered, however, by recipient immune recognition and rejection of major histocompatibility complex (MHC)-mismatched MSCs. Development of strategies to prevent immune rejection of MHC-mismatched MSCs *in vivo* is necessary to enhance cell survival and potentially increase the efficacy and safety of allogeneic MSC therapy. The purposes of this study were to evaluate if transforming growth factor-β2 (TGF-β2) downregulated MHC expression on equine MSCs and to determine if TGF-β2 treatment altered the phenotype of MSCs. Equine bone marrow-derived MSCs from 12 horses were treated with 1, 5, or 10 ng/ml TGF-β2 from initial isolation until MHC expression analysis. TGF-β2-treated MSCs had reduced MHC I and MHC II surface expression compared to untreated controls. TGF-β2 treatment also partially blocked IFN-γ-induced upregulation of MHC I and MHC II. Constitutive and IFN-γ-induced MHC I and MHC II expression on equine MSCs was dynamic and highly variable, and the effect of TGF-β2 was significantly dependent on the donor animal and baseline MHC expression. TGF-β2 treatment did not appear to change morphology, surface marker expression, MSC viability, or secretion of TGF-β1, but did significantly increase the number of cells obtained from culture. These results indicate that TGF-β2 treatment has promise for regulating MHC expression on MSCs to facilitate allogeneic therapy, but further work is needed to maintain MHC stability when exposed to an inflammatory stimulus.

## Introduction

Bone marrow-derived mesenchymal stem cell (MSC) therapy has shown significant promise for decreasing healing time and reducing reinjury rates in horses with musculoskeletal injuries (1). Although MSCs are capable of differentiating *in vitro*, the therapeutic properties of MSCs are derived primarily from immunomodulatory and trophic factors secreted by the cells (2–4). Allogeneic MSC therapy is particularly attractive because quality donor cells could be used at the time of injury. Currently, controversy exists over the use of autologous versus allogeneic MSCs. MSCs were originally described in early literature as immune-privileged due to their strong immunomodulatory properties (5). Analysis of alloimmune responses following *in vivo* transplantation have shown, however, that major histocompatibility complex (MHC)-mismatched MSCs do evoke both cell-mediated and humoral immune responses and do not persist as long as autologous MSCs (6–10). A recent study found that repeated intra-articular injections of allogeneic equine MSCs, but not autologous MSCs, resulted in adverse clinical signs, indicating an immune response against the allogeneic MSCs (11). We have also recently shown that horses injected with MHC-mismatched MSCs produce anti-MHC antibodies that are cytotoxic to MSCs as early as 7–14 days post transplantation (12, 13). Recipient immune recognition of mismatched MHC molecules on the surface of donor cells, termed allorecognition, and subsequent rejection of MSCs likely leads to decreased efficacy of therapy and an increase in the likelihood of adverse events.

Allorecognition of both MHC I and MHC II surface molecules on donor MSCs can contribute to *in vivo* rejection. MHC I molecules can be directly recognized by CD8^+^ T cells resulting in direct cytotoxicity of the foreign cell (14). CD4^+^ T cells can directly recognize MHC II surface molecules and enhance either cytotoxic or humoral immune responses. MHC molecules can also be shed by the donor cell and then internalized by antigen-presenting cells and presented to B or T cells, a process known as indirect recognition. Indirect recognition of MHC peptides is critical for CD4^+^ T cells to initiate class switching and alloantibody production in B cells (15, 16). Dendritic cells have been shown to present intact MHC I peptides to B cells, which leads to alloantibody generation (17). It is therefore critical to limit allorecognition of both MHC I and MHC II in order to prevent rejection of MHC-mismatched MSCs.

Development of strategies to limit allorecognition may help facilitate the use of allogeneic MSCs clinically. Downregulation of MHC surface expression is a common tactic utilized by viruses, neoplastic cells, and cells in immune-privileged tissues to avoid immune surveillance (18–20). In immune-privileged tissues like the brain, transforming growth factor-β2 (TGF-β2) is highly expressed and appears to help prevent autoantigen presentation and subsequent immune responses (21). TGF-β2 has been shown to downregulate constitutive MHC I surface expression on a variety of cell types including melanoma cells, intestinal epithelial cells, and astrocytes as well as block IFN-γ-induced MHC expression (22–24). The TGF-β2 isoform has previously been shown to be more effective than TGF-β1 at blocking IFN-γ-induced MHC expression (25). All equine MSCs express MHC I and can be heterogeneous for MHC II, but IFN-γ can strongly upregulate expression of MHC I and MHC II (26). Other inflammatory cytokines including IL-1β and TNF-α can alter MHC expression, but only IFN-γ upregulates both MHC I and MHC II surface expression (27). Because MSCs are typically injected directly into an inflammatory environment where IFN-γ may be present, it is particularly important to block IFN-γ-induced expression in order to limit allorecognition.

The aim of this study was to characterize the effects of TGF-β2 on constitutive and IFN-γ-induced MHC surface expression on equine bone marrow-derived MSCs. We also examined the morphology, cell surface marker expression, and TGF-β isoform secretion, which we define as the MSC phenotype, to determine if these were altered by TGF-β2 treatment.

## Materials and Methods

### Horses

A total of 12 horses were used for this study. All animals were between the ages of 5 and 17 years, free of systemic disease as determined by routine physical examinations and bloodwork, free of medication for 48 h prior to use, and non-pregnant. The Institutional Animal Care and Use Committee of North Carolina State University approved the use of horses in these studies.

### MSC Isolation and Culture

Bone marrow aspirates were collected aseptically from the sternum of 12 horses by using 11-gauge Jamshidi bone marrow biopsy needles under standing sedation with local anesthesia. For each harvest, a total of 120 ml of aspirate was collected into 60-ml syringes containing 25,000 U of heparin each. Bone marrow aspirates were purified *via* Ficoll-Paque Plus (GE Healthcare, Chicago, IL, USA) gradient centrifugation, as previously described (28). Isolated cells from each horse were evenly divided onto 100-mm tissue-culture plates containing the appropriate media for each treatment group. MSC base media used for the negative control group consisted of low glucose (1 g/dl) DMEM media containing 10% fetal bovine serum (FBS) (Atlanta Biologicals, Flowery Branch, GA, USA), 2 mM l-glutamine, penicillin (100 U/ml), and streptomycin (100 µg/ml). Standard MSC media used for the traditional control group consisted of MSC base media with the addition of basic fibroblastic growth factor (bFGF, 1 ng/ml) (Corning, Inc., Corning, NY, USA), which is a normal addition to media for equine MSC culture (12, 26). TGF-β2 treatment group media consisted of standard MSC media containing human recombinant TGF-β2 at concentrations of 1, 5, or 10 ng/ml (BioLegend, San Diego, CA, USA).

Media were exchanged every 48 h. Cells were passaged 1:3 at approximately 80% subconfluency by using Accumax cell-dissociation solution (Innovative Cell Technologies, Inc., San Diego, CA, USA) and plated at a density of approximately 1 × 10^4^ cells/cm^2^. Cell counts and viability at each passage were determined using a Cellometer^®^ Auto 2000 and ViaStain™ AOPI Staining Solution (Nexcelom Bioscience LLC, Lawrence, MA, USA). Cell cultures were imaged using an IX83 inverted microscope and cellSens™ software (Olympus Corporation, Center Valley, PA, USA). Cells to be cryopreserved were pelleted after dissociation and resuspended in freeze media (base or standard MSC medium with 10% FBS and 10% dimethyl sulfoxide).

### Immunophenotyping of MSCs

Mesenchymal stem cells were immunophenotyped at passage 2 (P2) for expression levels of MHC class I and MHC class II using FACS analysis. The MHC I and MHC II antibodies (clones cz3 and cz 11, respectively, Antczak Laboratory) used in this study were previously validated for the horse and used at dilutions of 1:10 according to previous experience (26, 28). Cells were pelleted in aliquots containing approximately 1 × 10^6^ cells on 96-well V-bottom plates and treated with a 20-min blocking step by using 10% normal goat serum in phosphate-buffered saline. The cells were pelleted and resuspended in unconjugated primary monoclonal antibody and incubated for 45 min at 4°C. MSCs were then washed and resuspended in a secondary allophycocyanin-conjugated goat anti-mouse IgG antibody (BD Biosciences, San Jose, CA, USA), and incubated for an additional 45 min at 4°C. Cells were analyzed on a LSRII (BD Biosciences) flow cytometer equipped with FACSDiva analysis software (BD Biosciences). MSCs stained with the secondary antibody alone were used as negative controls. Cells were gated as previously described and data were collected on a minimum of 1 × 10^4^ cells for each sample (26).

### MHC I Quantification

The QIFIKIT^®^ (Agilent Technologies, Inc., Carpinteria, CA, USA) was used to quantify MHC I surface expression in MSCs and equine fetal fibroblasts, which were isolated from a portion of the body wall of day 34 equine conceptuses collected by uterine lavage and grown to confluency. A F(ab′)_2_ fragment of FITC-conjugated goat anti-mouse IgG antibody from the kit was used as the secondary antibody for the setup beads, calibration beads, MSCs, and fetal fibroblasts. On an LSRII, the setup beads were used to set voltages and then the geometric mean fluorescence intensity (GMFI) was recorded for the five populations of calibration beads and MSCs. From the GMFIs of the calibration beads, a linear regression of the calibration curve was generated using using Prism 6 (GraphPad, La Jolla, CA, USA). Calculation of antigen binding capacity was completed from the linear regression according to the manufacturer’s instruction as a means of determining the average number of MHC I surface molecules for each MSC and fetal fibroblast population.

### IFN-γ Stimulation

**Figure 1:**
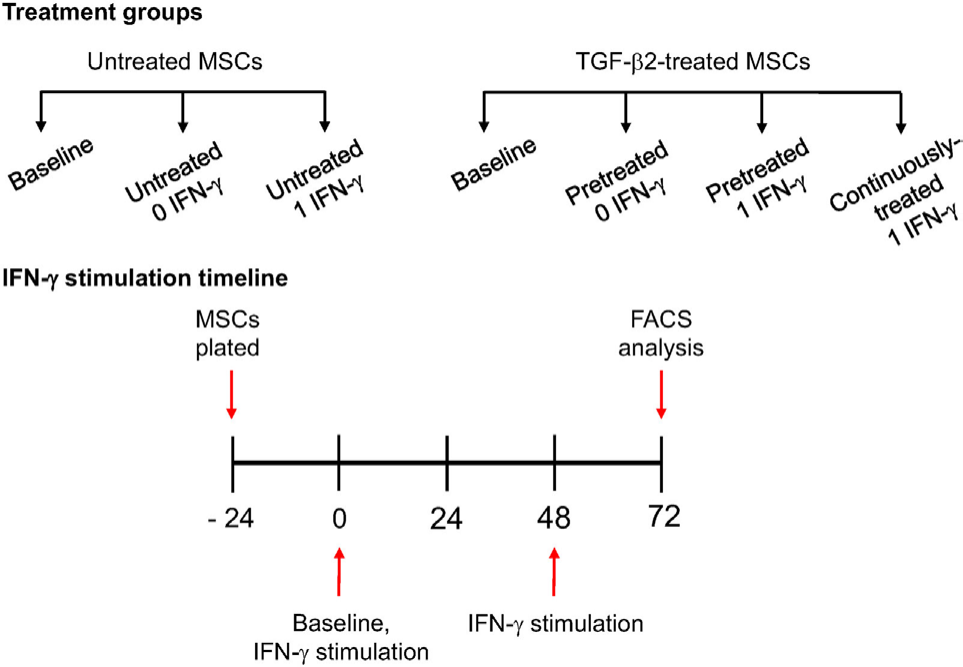
IFN-γ stimulation methods. P3 untreated [−/− transforming growth factor-β2 (TGF-β2)], pretreated (+/− TGF-β2), and continuously treated (+/+ TGF-β2) mesenchymal stem cells (MSCs) were stimulated with 1 ng/ml equine IFN-γ over a 72-h period. Untreated and pretreated MSCs not stimulated with IFN-γ were used as controls. Major histocompatibility complex (MHC) I and MHC II expression was measured *via* FACS following stimulation. Baseline MHC expression was obtained by freezing cells from each treatment group just prior to IFN-γ stimulation.

For immunophenotyping of IFN-γ-stimulated MSCs, P3 MSCs were divided into the following treatment groups: untreated MSCs (−/− TGF-β2), TGF-β2-pretreated MSCs (+/− TGF-β2), and continuously treated MSCs (+/+ TGF-β2) (Figure 1). Pretreated MSCs were cultured in TGF-β2 from initial isolation to just prior to IFN-γ stimulation. MSCs were stimulated by replacing cell culture media with standard media containing 1 ng/ml recombinant equine IFN-γ (R&D Systems, Minneapolis, MN, USA) at 0 and 48 h as shown in Figure 1. Untreated and pretreated MSCs not stimulated with IFN-γ were used as controls. Baseline MSC expression was determined by harvesting MSCs prior to IFN-γ stimulation. All 72 h MSC MHC I expression level, results were reported as fold change from that of the untreated (−/− TGF-β2) MSC group cultured without IFN-γ.

For IFN-γ-induced MHC expression kinetics, untreated and TGF-β2-pretreated P3 MSCs were stimulated with IFN-γ as described above. MSCs were harvested at baseline, 24, 48, and 72 h and frozen until FACS analysis.

### MSC Surface Markers

Cryopreserved TGF-β2-treated and untreated P2 and P3 MSCs were expanded for one passage before staining for surface markers as previously described (28). The following antibodies were used to confirm MSC phenotype: LFA-1 (cz3.2 Antczak lab), CD29 (TDM29 EMD Millipore), CD44 (CVS18 BioRad), CD45RB (DH16A Washington State University), and CD90 (DH24A Washington State University). LFA-1 was used neat, while dilutions of 1:10 (CD45RB), 1:100 (CD29, CD44), and 1:200 (CD90) were used, according to manufacturer’s directions and according to previous experience.

### Cytokine Analysis

P3 MSCs were divided into untreated and pretreated groups and stimulated with 1 ng/ml IFN-γ as described under IFN-γ stimulation. All groups were washed at 72 h and media replaced with standard media containing no IFN-γ or TGF-β2. Supernatant was collected 24 h later for analysis of TGF-β1, 2, and 3 using a commercially available multiplexed cytokine/chemokine magnetic bead kit (EMD Millipore, Billerica, MA, USA). All samples were analyzed in duplicate using a 96-well platform performed per manufacturer’s instructions. Sample volume used was 25 µl and all samples were run at a 1:5 dilution. Plates were read using a Luminex MagPix instrumentation (Luminex Corporation, Austin, TX, USA). A minimum bead count of 50 for each cytokine was acquired for data analysis. Data were analyzed using Milliplex Analyst 5.1 software (Luminex Corporation, Austin, TX, USA).

### Statistical Analyses

Immunophenotyping and IFN-γ stimulation data were normalized by log transformation and analyzed by analysis of covariance (ANCOVA) with horse as covariate, followed by the Tukey’s test for multiple comparisons. TGF-β concentrations in supernatant were analyzed by ANCOVA with horse as covariate, followed by the Tukey’s test for multiple comparisons. Baseline MHC data for the IFN-γ stimulation experiments, cell yield, and viability were analyzed using a two-tailed *t*-test. Analyses were performed using JMP^®^ Pro11 (SAS Institute Inc., Cary, NC, USA) and significance set at *p* < 0.05.

## Results

### MSC Immunophenotyping

**Figure 2:**
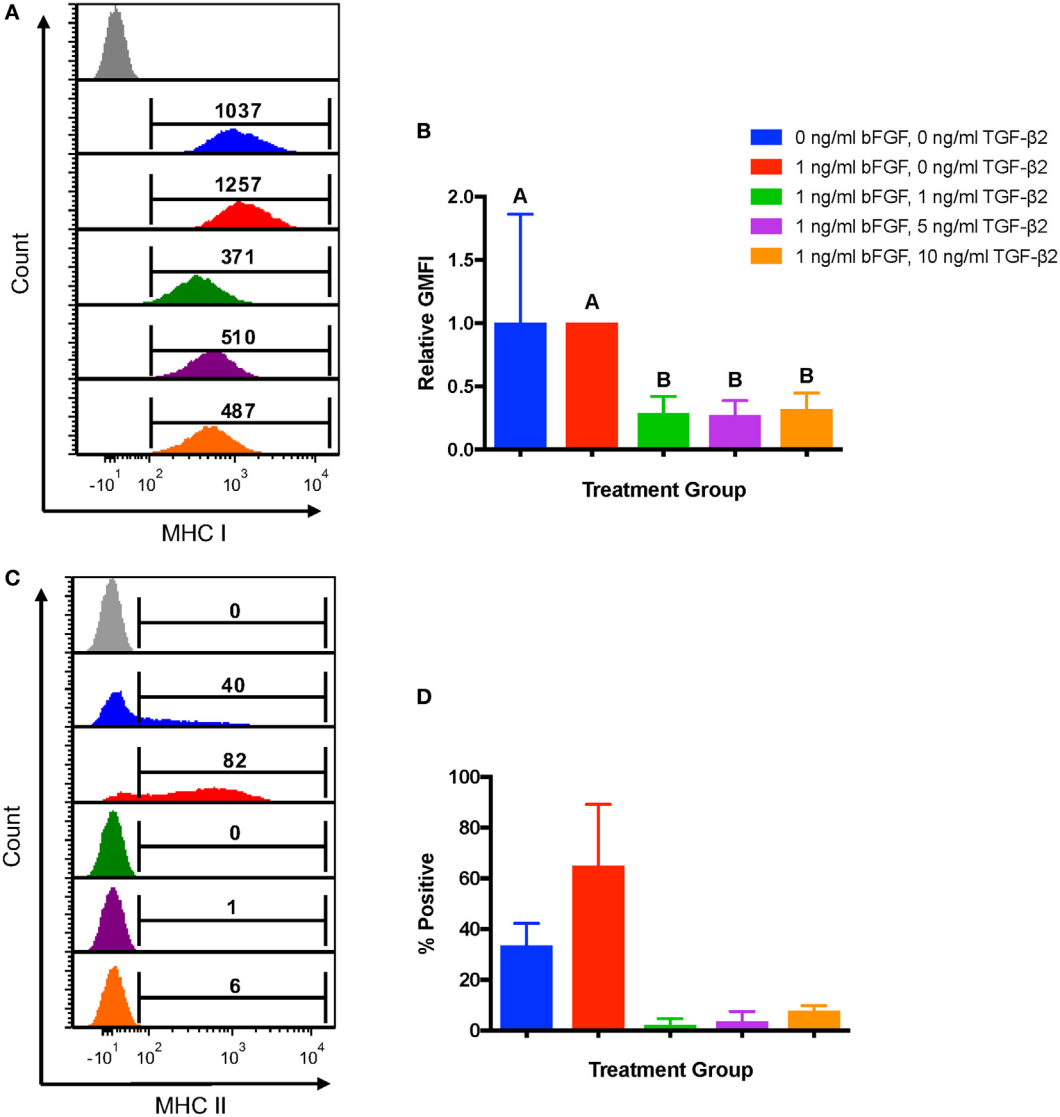
Major histocompatibility complex (MHC) I and MHC II surface expression on untreated and transforming growth factor-β2 (TGF-β2)-treated mesenchymal stem cells (MSCs). Equine MSCs were cultured in media containing 0 ng/ml basic fibroblastic growth factor (bFGF) and 0 ng/ml TGF-β2 (negative control group), 1 ng/ml bFGF and 0 ng/ml TGF-β2 (traditional control group) or 1 ng/ml bFGF and 1, 5, or 10 ng/ml TGF-β2 from initial isolation to P2. MHC I and MHC II expression was measured *via* FACS analysis. Superscript letters indicate significant differences between groups. **(A)** Representative histograms from one horse of MHC I expression for all treatment groups. Numbers above gates represent population geometric mean fluorescence intensity (GMFI) compared to MHC I negative control (gray). **(B)** MHC I expression shown as the average fold change in GMFI relative to the traditional control group. Data shown are mean ± SD of *n* = 8, *p* < 0.0001. **(C)** Representative histograms from one horse of MHC II expression for all treatment groups. Numbers above gates represent the percent of the parent population positive for MHC II compared to the negative control. **(D)** MHC II expression shown as the average percent of MSCs positive for MHC II. Data shown are mean ± SD of *n* = 2.

To first determine the effects of TGF-β2 treatment on MSC immunophenotype, MSCs from the same bone marrow aspirate were cultured with 1, 5, or 10 ng/ml TGF-β2. Untreated MSCs cultured in standard media with or without bFGF were used as controls. GMFI was used to assess expression levels of MHC I because all MSCs should be positive for MHC I with a fairly normal distribution of expression. MSCs cultured in 1, 5, and 10 ng/ml TGF-β2 all had lower MHC I surface expression compared with either control group (Figure 2A) and had a significantly lower fold change in GMFI compared to the traditional control group (Figure 2B). There was no significant difference in MHC I expression between any of the TGF-β2 concentrations. Negative control MSCs (0 ng/ml bFGF) had the largest variation in MHC I expression, but the relative GMFI was not significantly different from the traditional control group (1 ng/ml bFGF) indicating that bFGF may have some stabilizing effect on MHC I expression.

Major histocompatibility complex II expression was reported as the average percent of cells positive for each control and treatment group as expression can be heterogeneous and not accurately represented by any measure of MFI. MSCs from two of the eight horses sampled for this experiment were positive for MHC II. Treatment with all concentrations of TGF-β2 strongly reduced the proportion of P2 MSCs positive for MHC II, but statistical significance could not be determined due to the low sample size (Figures 2C–D).

### MHC I Quantification

**Figure 3:**
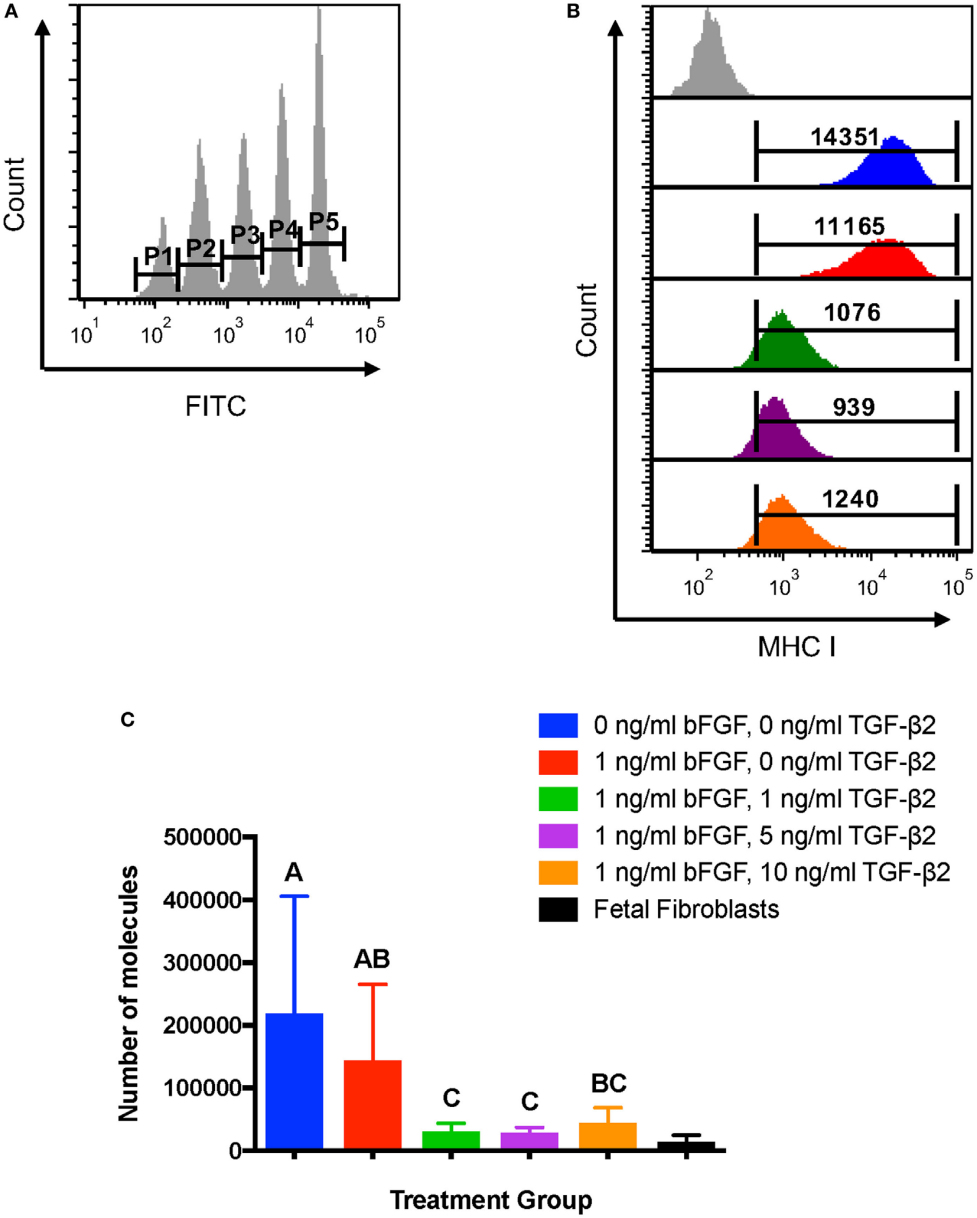
Quantification of major histocompatibility complex (MHC) I surface expression on untreated and transforming growth factor-β2-treated mesenchymal stem cells (MSCs). The QIFIKIT^®^ assay was used to quantify the average number of MHC I surface molecules on passage 2 MSCs based on the population geometric mean fluorescence intensity (GMFI). Superscript letters indicate significant differences between groups. **(A)** Calibration bead populations were gated to obtain GMFI for linear regression analysis. **(B)** Representative histograms from one horse showing MHC I expression and population GMFI for all treatment groups. **(C)** Average number of MHC I molecules as determined by linear regression analysis for all treatment groups. Data shown are mean ± SD of *n* = 7, *p* = 0.0003. Fetal fibroblasts are included as a MHC I^low^ reference cell.

Average MHC I surface expression was quantified for each treatment group using the Dako QIFIKIT^®^ to determine the number of surface molecules expressed by MSCs. Quantification of MHC I allows for direct comparison of expression to fetal fibroblasts, which are considered non-immunogenic in part due to low MHC I surface expression (29). The GMFI of each calibration bead population was obtained (Figure 3A) and used to generate a linear regression equation for antigen binding capacity. The average MHC I GMFI of each MSC treatment group for each horse was then used to calculate the average antigen binding capacity, which would correlate in this experiment to the number of MHC I surface molecules (Figure 3B). The assay confirmed that P2 MSCs treated with TGF-β2 have significantly fewer MHC I surface molecules than MSCs from either control group (Figure 3C). MHC I expression levels of treated cells were also more similar to equine fetal fibroblasts than control groups and had less variation in expression between horses (Figure 3C). Due to the small number of fetal fibroblast samples (*n* = 3), fetal fibroblast MHC I expression was not included in the statistical analysis.

As there were no significant differences between MHC I expression for the three TGF-β2 concentrations, the lowest concentration, 1 ng/ml, was used for all subsequent experiments. The traditional control group, which was cultured with bFGF, was also used as the untreated control for all other experiments.

### IFN-γ Stimulation

**Figure 4:**
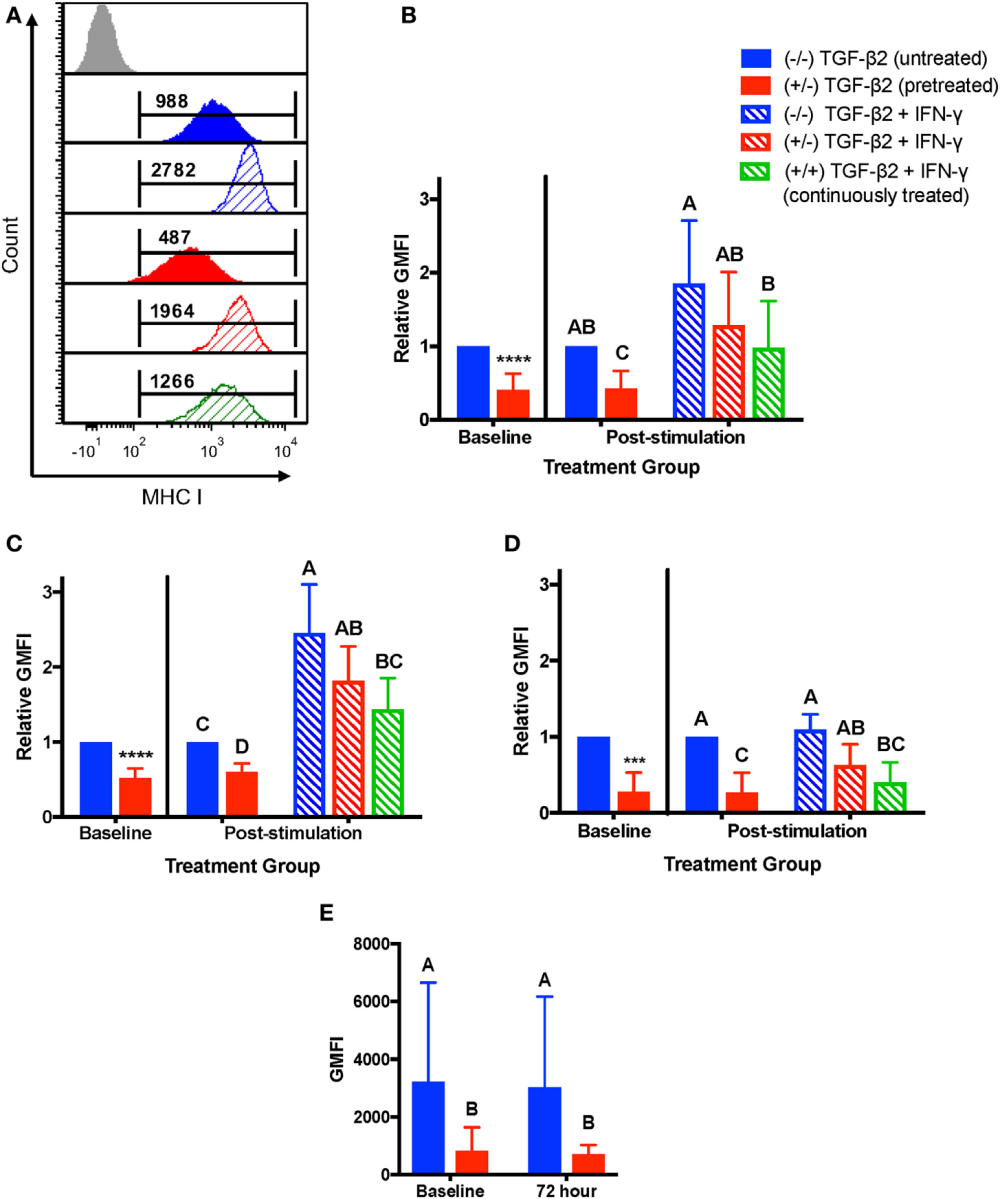
Major histocompatibility complex (MHC) I surface expression on untreated and transforming growth factor-β2 (TGF-β2)-treated mesenchymal stem cells (MSCs) following IFN-γ stimulation. P3 untreated (−/−), TGF-β2-pretreated (+/−), and TGF-β2 continuously treated (+/+) MSCs were stimulated with 1 ng/ml IFN-γ for 72 h before MHC I expression analysis *via* FACS. Untreated and pretreated MSCs not stimulated with IFN-γ were used as controls. Superscript letters indicate significant differences between groups. **(A)** Representative histograms from one horse of MHC I expression for all treatment groups. Numbers above gates represent population geometric mean fluorescence intensity (GMFI) compared to MHC I negative control (gray). **(B)** MHC I expression shown as the average fold change in GMFI relative to the control group (−/− TGF-β2 untreated, solid blue bar). Data shown are mean ± SD of *n* = 9, *p < 0.0001 by *t*-test, *p* < 0.0001 by analysis of covariance (ANCOVA). **(C)** MHC I expression for MHC I low horses shown as the average fold change in GMFI relative to the control group (−/− TGF-β2 untreated, solid blue bar). Data shown are mean ± SD of *n* = 5, *p < 0.0001 by *t*-test, *p* < 0.0001 by ANCOVA. **(D)** MHC I expression for MHC I high horses shown as the average fold change in GMFI relative to the control group (−/− TGF-β2 untreated, solid blue bar). Data shown are mean ± SD of *n* = 4, *p = 0.0049 by *t*-test, *p* < 0.0001 by ANCOVA. **(E)** MHC I GMFI of baseline and unstimulated MSCs treatment groups. Data shown are mean ± SD of *n* = 9, *p* < 0.0001.

As MSCs are typically injected directly into sites of active inflammation, we measured the stability of MHC I and MHC II surface expression on untreated, TGF-β2-pretreated, and TGF-β2 continuously treated MSCs following stimulation with IFN-γ. IFN-γ stimulation induced upregulation of MHC I surface expression on untreated and TGF-β2-treated cells as compared to the unstimulated controls (Figure 4A). Relative to the unstimulated and untreated control group, there was no significant difference in MHC I expression in any of the treatment groups following IFN-γ stimulation, although the pretreated and continuously treated MSCs did trend toward having lower MHC I expression (Figure 4B). However, the continuously treated TGF-β2 MSCs did have significantly lower MHC I expression than the untreated controls following IFN-γ stimulation, indicating that TGF-β2 is able to partially block IFN-γ-induced MHC I expression under these conditions. There was a significant variation in MHC I expression between horses that affected how strongly the cells upregulated MHC I with IFN-γ stimulation. Individual horses could be divided into two categories, MHC I low and MHC I high, depending on the fold change in MHC expression following IFN-γ stimulation. Horses that had at least a 1.8-fold change increase in expression following IFN-γ stimulation and also had the lowest MHC I expression (Figure 4C). Horses that already expressed high levels of MHC I had less than a 1.4-fold change increase in expression (Figure 4D). Over the 72 h without TGF-β2, MHC I expression of pretreated MSCs not stimulated with IFN-γ was not significantly different from baseline expression and still significantly lower than untreated MSCs (Figure 4E).

**Figure 5:**
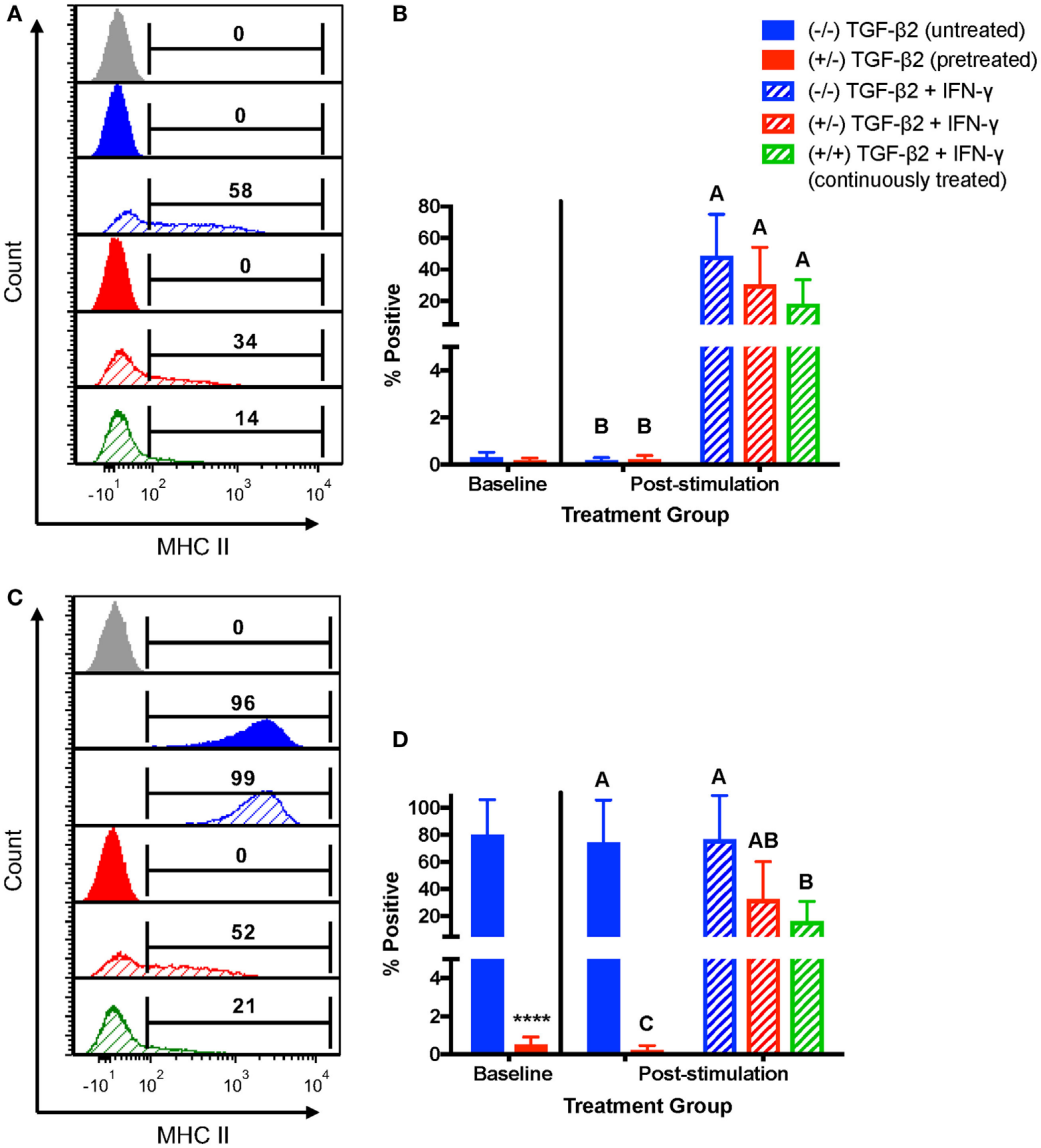
Major histocompatibility complex (MHC) II surface expression on untreated and transforming growth factor-β2 (TGF-β2)-treated mesenchymal stem cells (MSCs) following IFN-γ stimulation. P3 untreated (−/−), TGF-β2-pretreated (+/−), and TGF-β2 continuously treated (+/+) MSCs were stimulated with 1 ng/ml IFN-γ for 72 h before MHC II expression analysis *via* FACS. Untreated and pretreated MSCs not stimulated with IFN-γ were used as controls. Superscript letters indicate significant differences between groups. **(A)** Representative histograms from one MHC II baseline negative horse showing MHC II expression for all treatment groups. Numbers above gates represent the percent of the parent population positive for MHC II compared to the negative control (gray). **(B)** MHC II expression shown as the average percent of the parent population positive for MHC II relative to the control group (−/− TGF-β2 untreated, solid blue bar). Data shown are mean ± SD of *n* = 6, *p* < 0.0001. **(C)** Representative histograms from one MHC II baseline positive horse showing MHC II expression for all treatment groups. Numbers above gates represent the percent of the parent population positive for MHC II compared to the negative control (gray). **(D)** MHC II expression shown as the average percent of the parent population positive for MHC II relative to the control group (−/− TGF-β2 untreated, solid blue bar). Data shown are mean ± SD of *n* = 3, *p < 0.0001 by *t*-test, *p* < 0.0007 by analysis of covariance.

For MHC II, horses were characterized as MHC II negative or MHC II positive depending on baseline expression. Untreated MSCs from four horses were negative for MHC II and untreated MSCs from three horses were positive for MHC II at the beginning of the IFN-γ stimulation. For MHC II negative MSCs, neither pretreatment nor continuous treatment significantly prevented IFN-γ-induced expression of MHC II (Figures 5A,B), although like MHC I, treated MSCs tended to have a lower number of MSCs positive for MHC II. For MSCs that were MHC II positive prior to IFN-γ challenge, only the MSCs continuously treated with 1 ng/ml TGF-β2 had significantly fewer cells with MHC II expression compared with untreated MSCs (Figures 5C,D). Significant upregulation still occurred, however, compared with TGF-β2-treated MSCs that were not challenged with IFN-γ again indicating partial, but incomplete, blocking of IFN-γ-induced MHC expression.

### IFN-γ-Induced MHC Kinetics

**Figure 6:**
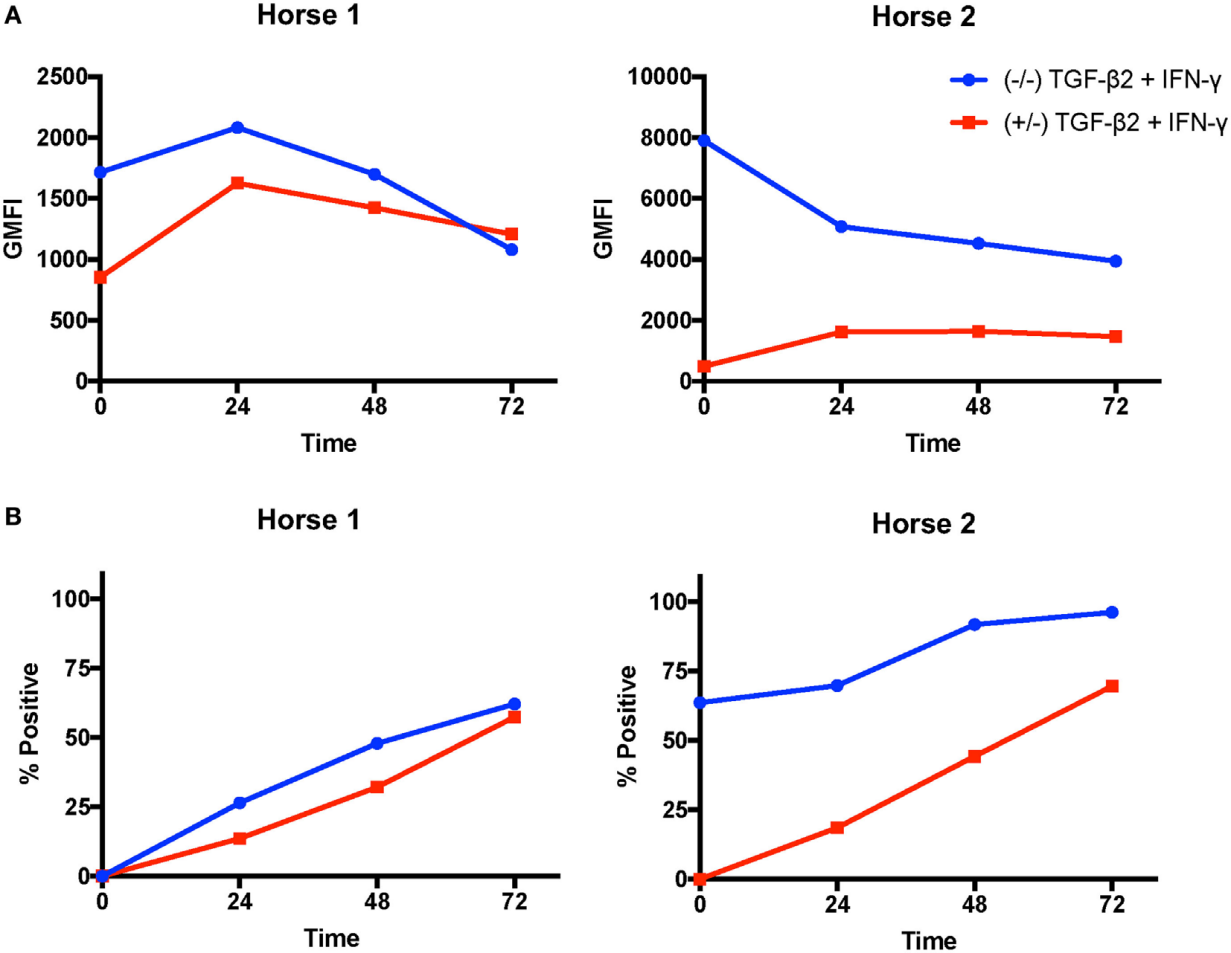
Major histocompatibility complex (MHC) I and MHC II surface expression kinetics on untreated and transforming growth factor-β2 (TGF-β2)-pretreated mesenchymal stem cells (MSCs) following IFN-γ stimulation. MHC I and MHC II expression were measured on untreated and TGF-β2-pretreated MSCs from two horses *via* FACS every 24 h for 72 h following IFN-γ stimulation. **(A)** MHC I expression shown as the geometric mean fluorescence intensity (GMFI) of MSCs in each treatment group over time. **(B)** MHC II expression shown as the percent of the parent MSC population positive for MHC II in each treatment group over time.

Next, we analyzed if TGF-β2 treatment changed the rate of IFN-γ-induced MHC I and MHC II upregulation. MSCs from two horses were compared in this assay. On MSCs from Horse 1, MHC I expression peaked at 24 h on both untreated and pretreated MSCs, then declined over the next 48 h (Figure 6A). Pretreated MSCs had lower MHC I expression at 0, 24, and 48 h, but slightly higher expression at 72 h. Horse 2 had very high MHC I baseline expression on untreated MSCs and expression decreased over the 72 h (Figure 6A). Horse 1 was baseline MHC II negative and Horse 2 was baseline MHC II positive. MHC II expression on MSCs from both horses gradually increased throughout the 72 h of the assay in both untreated and pretreated MSCs (Figure 6B). MHC II expression on pretreated MSCs remained lower than untreated MSCs throughout the assay. The MHC I expression patterns at 72 h were different in these two individual horses than what was seen overall in the previous IFN-γ stimulation assay further demonstrating that MHC I expression on MSCs is dynamic and dependent on baseline expression and the individual MSC donor.

### MSC Morphology and Surface Markers

**Figure 7:**
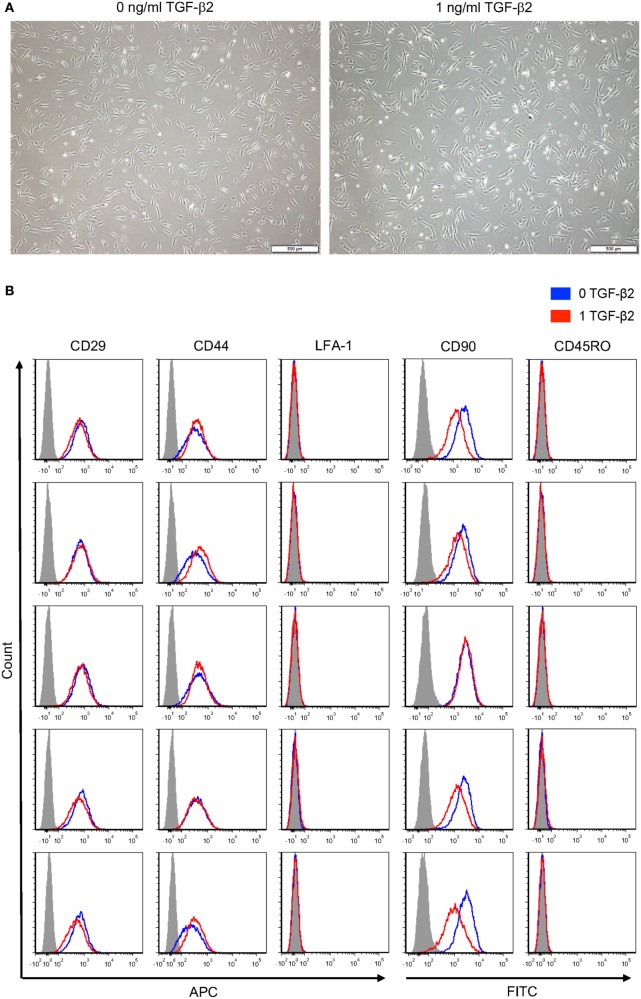
Phenotype of untreated and transforming growth factor-β2 (TGF-β2)-treated mesenchymal stem cells (MSCs). **(A)** P2 MSCs were imaged *via* phase microscopy, bar = 500 µM. **(B)** P3 untreated and TGF-β2-treated MSCs were stained for positive MSC surface markers CD29, CD44, and CD90 and negative surface markers LFA-1 and CD45RO. Expression is shown as FACS histograms from five horses.

Morphology of P2 untreated and TGF-β2-treated MSCs was visually compared using phase contrast microscopy. Untreated and TGF-β2-treated MSCs were both spindle shaped with similar width and length (Figure 7A). Expression of standard MSC surface markers was measured to determine if 1 ng/ml TGF-β2 treatment altered equine MSC surface marker expression. Both untreated and TGF-β2-treated MSCs were positive for CD29, CD44, and CD90 and negative for CD45RO and LFA-1 (Figure 7B). Overall, there were not significant differences between the expression for treated or untreated MSCs for any of the positive or negative surface markers, except CD90 expression was slightly downregulated on TGF-β2-treated MSCs in four of the five horses. Unlike MHC surface expression, expression levels of CD29, CD44, and CD90 were very similar between individual horses.

### Cell Yield and Viability

**Figure 8:**
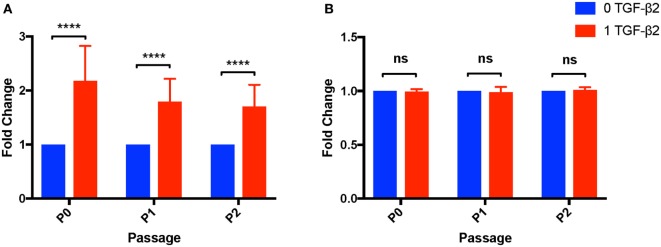
Cell yield and viability of untreated and transforming growth factor-β2 (TGF-β2)-treated mesenchymal stem cells (MSCs). Cell yield and viability were determined at each passage using a Cellometer^®^ Auto 2000 and ViaStain™ AOPI Staining Solution. **(A)** Cell yield is displayed as the average fold change in cell counts relative to untreated MSCs. Data shown are mean ± SD of *n* = 8, ****p < 0.0001 by *t*-test **(B)** Viability is displayed as the average fold change in percent viability of MSCs relative to untreated MSCs. Data shown are mean ± SD of *n* = 8.

Transforming growth factor-β2 is well known as a negative regulator of the cell cycle and can induce apoptosis in certain cell types (30, 31). To determine effects of TGF-β2 treatment on MSC proliferation capacity, the number and viability of cells obtained from P0 to P2 was compared between untreated and TGF-β2-treated MSCs. For P0–P2, TGF-β2-treated MSCs had significantly increased cell yields compared to untreated, despite there the fact that the same number of cells were plated at the beginning of each passage and the groups were grown for the same amount of time (Figure 8A). The relative difference between cell yield appeared to diminish with each passage, but there was still a significantly higher cell yield at P2 in the TGF-β2-treated group. The increase in cell yield appears to be dependent on coculture with bFGF as MSCs grown with only TGF-β2 had significantly reduced cell yield relative to cells cultured with bFGF and TGF-β2 (*p* < 0.0001) (Figure S1 in Supplementary Material). There was no significant difference between the viability of untreated or treated MSCs from P0 to P2 (P0: *p* = 0.257, P1: *p* = 0.313, P2: *p* = 0.100) (Figure 8B).

### TGF-β Isoform Production

**Figure 9:**
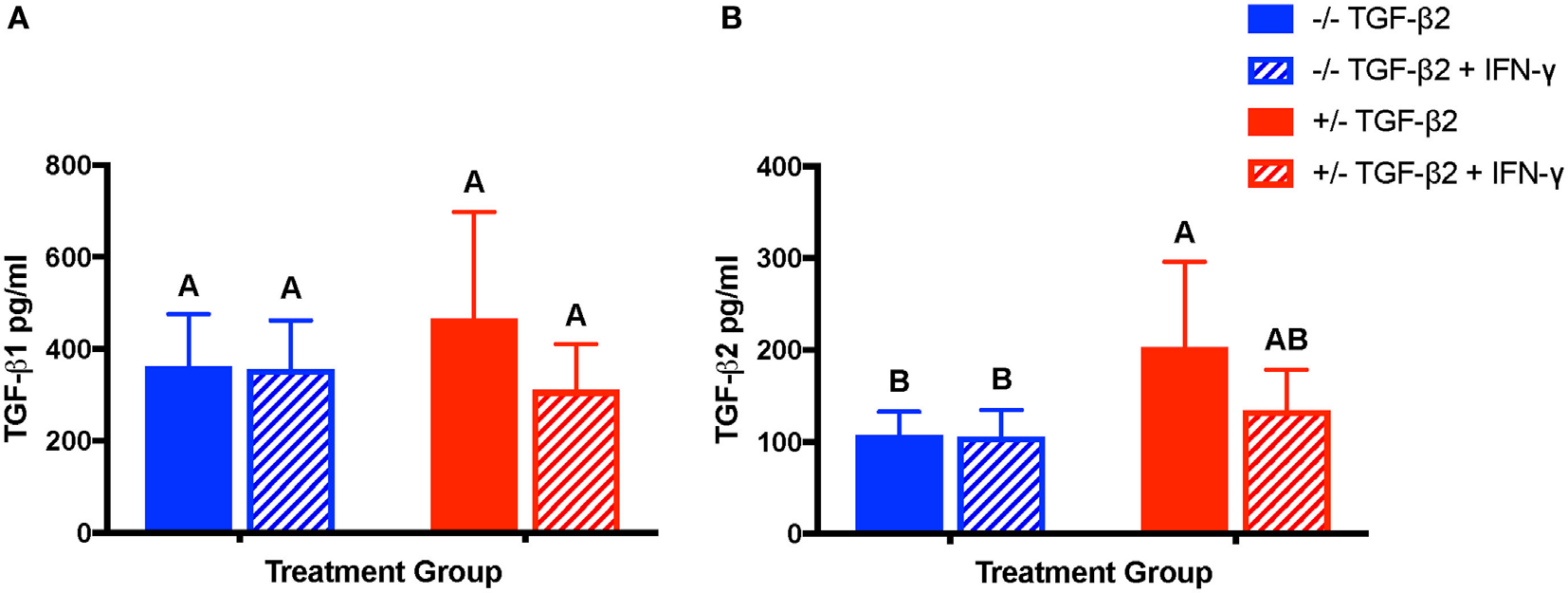
Production of TGF-β1 and transforming growth factor-β2 (TGF-β2) by untreated and TGF-β2-treated mesenchymal stem cells (MSCs). TGF-β1 and TGF-β2 concentrations were measured in the supernatant of untreated (−/− TGF-β2), untreated and IFN-γ stimulated (−/− TGF-β2 + IFN-γ), TGF-β2-pretreated (+/− TGF-β2), and TGF-β2-pretreated and IFN-γ stimulated (+/− TGF-β2 + IFN-γ) MSCs using a TGF-β multiplex assay. Superscript letters indicate significant differences between groups. **(A)** TGF-β1 is displayed as the mean concentration ± SD of *n* = 7. **(B)** TGF-β2 is displayed as the mean concentration ± SD of *n* = 7, *p* = 0.0045 by analysis of covariance.

Constitutive secretion of TGF-β1, but not TGF-β2, has been previously reported from equine MSCs (32). Concentrations of TGF-β1 and TGF-β2 were measured in the supernatant of unstimulated and IFN-γ-stimulated MSC cultures to determine if treatment with TGF-β2 affected production of either TGF-β isoform. Unstimulated and IFN-γ-stimulated MSCs from both untreated and TGF-β2-pretreated groups secreted TGF-β1 as well as TGF-β2 (Figures 9A,B). There was no significant difference between the concentrations of TGF-β1 secreted by untreated or TGF-β2-pretreated MSCs or between unstimulated and stimulated MSCs (*p* = 0.102) (Figure 9A), but unstimulated TGF-β2-pretreated MSCs did secrete significantly more TGF-β2 than untreated MSCs (Figure 9B). Expression of TGF-β1 was significantly different between individual horses (*p* = 0.011). TGF-β3 could not be detected in any of the samples (data not shown).

## Discussion

The aims of this study were to determine the effects of TGF-β2 on MHC expression, phenotype, and TGF-β isoform secretion of equine bone marrow-derived MSCs. The data obtained in this study are consistent with these previous findings in other cell types that TGF-β2 is capable of downregulating MHC I and II expression and partially blocking IFN-γ-induced expression (22–25). Our studies also confirm that MSCs treated with TGF-β2 retain their phenotype and are able to secrete high levels of the immunomodulatory cytokine TGF-β1 as well as TGF-β2.

As shown in our initial immunophenotyping, although all MSCs are positive for MHC I expression, the degree of MHC I expression on untreated bone marrow-derived MSCs is highly variable between individual horses. MSCs treated with TGF-β2 in this study had consistently lower MHC I surface expression than untreated MSCs, less variation in MHC I expression levels than untreated MSCs, and similar expression levels as fetal fibroblasts. It is important to note that the TGF-β2-treated cells are still positive for MHC I, as absent MHC I expression would result in natural killer cell-mediated lysis (33). As shown in the MHC I quantification assay, constitutive MHC I expression levels on MSCs are highly variable between horses, but in our experience, individual horses tend to have consistent MHC I expression levels between bone marrow aspirates. This variation between individuals has also been documented in humans and chickens and is influenced by MHC haplotype in chickens (34, 35). The immunophenotyping results for MHC II expression on untreated MSCs are consistent with previous findings that equine bone marrow-derived MSCs are heterogeneous for MHC II expression (26). Untreated MSCs from two of the initial eight horses used for testing the three TGF-β2 concentrations were positive for MHC II, but treatment with TGF-β2 was able to dramatically reduce MHC II expression on unstimulated MSCs to nearly undetectable levels. Further research on individual variance in MHC I and MHC II expression levels may be helpful for identifying ideal donor horses for allogeneic MHC therapy and for developing further strategies to regulate MHC expression.

Similar to a previous study on intestinal epithelial cells (23), IFN-γ-induced upregulation of MHC I on MSCs in this study was attenuated by TGF-β2 treatment, although the significance was dependent on if the donor horse was MHC I baseline high or low. The degree to which IFN-γ was able to upregulate MHC I and TGF-β2 to block IFN-γ-induced MHC I expression appeared to be highly dependent on the individual horse from which the MSCs were obtained. It is still unclear if these differences in response to IFN-γ and TGF-β2 between horses are influenced by MHC haplotype or the health status of the animal, although all horses in this study were systemically healthy. TGF-β2 treatment also reduced the proportion of MSCs positive for MHC II following IFN-γ challenge, but this change was only significant for MSC populations that were already positive for MHC II at baseline measurement. Continuous treatment with TGF-β2 was shown to be the most effective at blocking IFN-γ-induced MHC I and MHC II expression in MSCs and co-delivery of TGF-β2 *via* encapsulation in hydrogels or scaffolds with MSCs may be possible for *in vivo* therapy (36). Other methods that can be used with TGF-β2 treatment expression, like 3D culture (37) or manipulation of expression of the class II transactivator (38), should also be explored to stabilize MHC expression on MSCs.

In comparison to the changes seen in MHC expression, TGF-β2 treatment did not appear to significantly change the morphology, surface markers, or viability of MSCs. TGF-β2-treated MSCs were still positive for MSC surface markers CD29, CD44, and CD90 and negative for hematopoietic markers CD45RO and LFA-1. Four of the five horses had reduced CD90 expression, but CD90 has previously been shown to have no effect on the immunomodulatory properties of human MSCs (39). TGF-β2 treatment also had unexpected, yet positive effects on the proliferative capacity of MSCs. TGF-β isoforms are well understood to negatively regulate of cell cycle progression from G1 to S phase (30), but treatment of MSC cultures with 1 ng/ml TGF-β2 significantly increased the cell yield of early passage MSCs in this study. TGF-β has been shown to increase production of connective tissue growth factor, a mitogenic peptide, in fibroblasts (40) and may have similar effects on production in MSCs. MSCs from older patients may not proliferate as well as younger and healthier patients (41, 42), so TGF-β2 treatment may be a viable option for increasing autologous MSC cell yield from older horses or horses with poorly proliferating MSCs. It is important to note that MSCs treated with TGF-β2 in the absence of bFGF have reduced proliferative abilities compared to MSCs cultured with both indicating that cotreatment with bFGF is necessary. The difference in proliferative capabilities depending on the presence of bFGF may be due to changes in cell cycle progression and should be explored in future studies. The effects of TGF-β2 treatment on the *in vitro* differentiation capabilities or other paracrine factors were not explored in this study, but may be relevant for future therapies. As TGF-β isoforms activate transcription factors with broad cellular effects (43), it is possible that treatment may alter the potency or therapeutic properties of the cells (44).

We also report that equine MSCs constitutively express TGF-β1 and TGF-β2 and that TGF-β2 treatment does not negatively impact expression of either of these. *In vitro* assays like mixed leukocyte reactions and cytotoxicity assays are needed to determine if the modest increases in TGF-β2 expression seen in TGF-β2-treated MSCs change the immunomodulatory properties of the cells and immune recognition. There was also significant variation in concentrations of TGF-β1 and TGF-β2 secreted by individual horses further supporting that some animals may make more ideal donors based on the MHC expression and cytokine profiles of their cells.

In conclusion, TGF-β2 downregulates constitutive MHC I and MHC II surface expression, partially blocks the effects of IFN-γ on MHC expression, and does not significantly alter the morphology, cell surface marker expression, or secretion of TGF-β1 in equine bone marrow-derived MSCs. These findings warrant further investigation into the *in vitro* and *in vivo* cell-mediated immunogenicity of TGF-β2-treated MSCs to determine if downregulation of MHC I and MHC II expression is sufficient to prevent immune rejection of MHC-mismatched MSCs and to improve clinical applications.

## Ethics Statement

The Institutional Animal Care and Use Committee of North Carolina State University approved the use of horses in these studies.

## Author Contributions

We certify that all authors meet the qualifications for authorship as listed below: (1) substantial contributions to conception or design of the work or the acquisition, analysis, or interpretation of data for the work; (2) drafting the work or revising it critically for important intellectual content; (3) final approval of the version to be published; (4) agreement to be accountable for all aspects of the work in ensuring that questions related to the accuracy or integrity of any part of the work are appropriately investigated and resolved.

## Conflict of Interest Statement

The authors declare that the research was conducted in the absence of any commercial or financial relationships that could be construed as a potential conflict of interest.

## Supplementary Material

Figure S1 Effects of basic fibroblastic growth factor (bFGF) and transforming growth factor-β2 (TGF-β2) on cell yield. Cell yield was determined by manual hand count. Cell yield is displayed as the average fold change in cell count relative to mesenchymal stem cells cultured with 1 ng/ml bFGF, 5 ng/ml TGF-β2. Data shown are mean ± SD of *n* = 6, *****p* < 0.0001 by *t*-test.
