# Supplementary figures and images for: Transforming Growth Factor-β2 Downregulates Major Histocompatibility Complex (MHC) I and MHC II Surface Expression on Equine Bone Marrow-Derived Mesenchymal Stem Cells Without Altering Other Phenotypic Cell Surface Markers
Source: Front Vet Sci. 2017 Jun 12;4:84. doi: 10.3389/fvets.2017.00084 (PMC5466990; doi:10.3389/fvets.2017.00084)

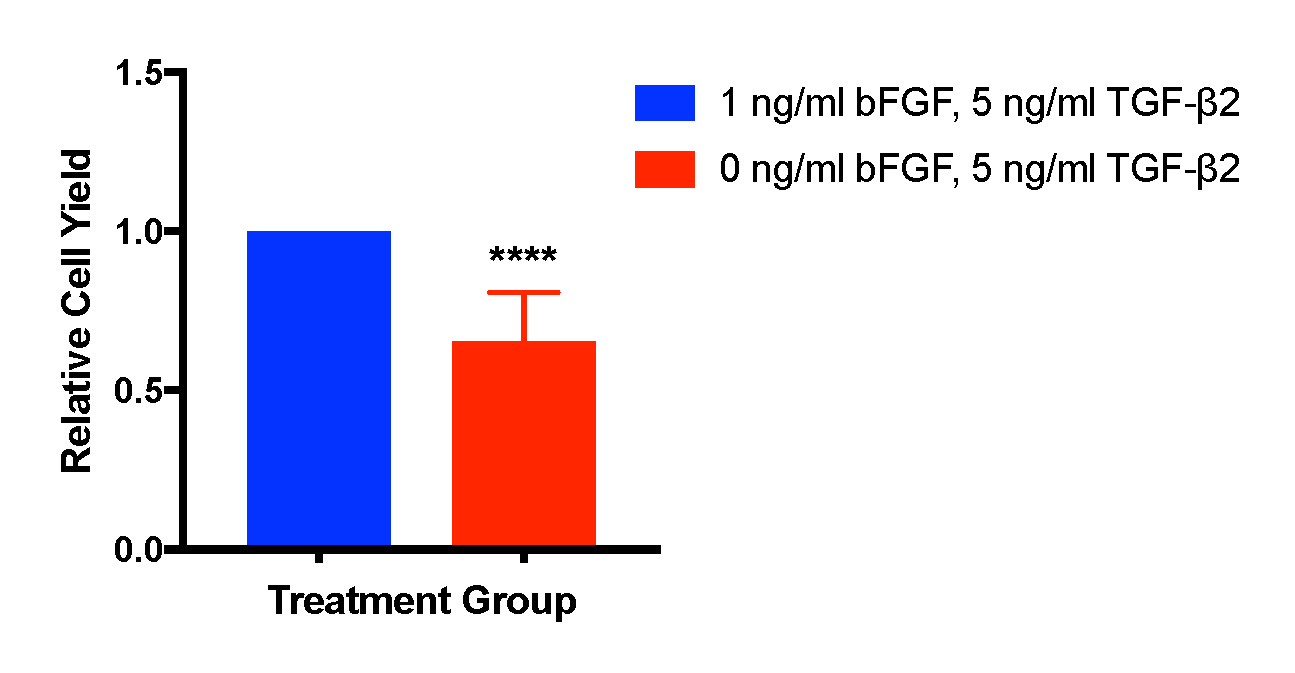

Supplement: Figure S1 — Effects of basic fibroblastic growth factor (bFGF) and transforming growth factor-β2 (TGF-β2) on cell yield. Cell yield was determined by manual hand count. Cell yield is displayed as the average fold change in cell count relative to mesenchymal stem cells cultured with 1 ng/ml bFGF, 5 ng/ml TGF-β2. Data shown are mean ± SD of n = 6, ****p < 0.0001 by t-test. [file image_1.tiff]
